# Integrating Health and Social Services in Finland: Regional Approaches and Governance Models

**DOI:** 10.5334/ijic.5982

**Published:** 2022-09-14

**Authors:** Hanna Tiirinki, Juhani Sulander, Timo Sinervo, Saija Halme, Ilmo Keskimäki

**Affiliations:** 1 Department of Social Research, University of Turku, Finland; 2 Welfare State and Reform Unit, Finnish Institute for Health and Welfare, THL, Finland; 3 Administration Department, Data Governance Unit, National Supervisory Authority for Welfare and Health, Finland; 4 Health Services Research, Faculty of Social Sciences, Tampere University, Finland

**Keywords:** integration, integrated care, social and health care, governance

## Abstract

**Introduction:**

The study explores regional approaches to integrated care, focusing on regions with regular municipality-based and integrated unified health and social care administration. The aim is to describe a governance approach that supports care integration in the regions.

**Methods:**

The study draws on analysis of integrated care governance using an extensive collection of administrative documents (*n* = 176) on regional health and social services within 20 specialised care authorities. The document data were supplemented with interviews of national health and social system evaluation officers. In our analysis, we used deductive content analysis and identified conceptual approaches of social and health care integration according to elements of integrated care governance.

**Results:**

Overall, integrated care governance was relatively well advanced. All regional authorities had established at least some preconditions for integrated governance. The stage of integration varied in the different elements of integrated care governance. The regions with unified integrated administrations enabled the more advanced models of integrated care.

**Conclusions:**

Various models for cooperation between regional health and social care authorities have emerged in the regions to identify good integrated care practices. The study suggests that the applied theoretical framework and presented elements of integrated care governance can be used to monitor development of care integration.

## Introduction

The social and health system – and the degree to which it functions as it was designed – plays a critical role in promoting, restoring, and maintaining people’s health and well-being. In several countries, including Finland, health and social care systems have been reoriented to embrace an integrated, people-centred approach to improve quality, people’s experience, and sustainability of the service systems [1,2]. In Finland, an advanced integrated social and health service system has been introduced as the main goal for reforming the system by several subsequent governments [3,4,5].

The need for better interplay and processes between various sectors of health and social services that are more fluent is evident. First, demographic and epidemiological changes, the rising expectations of the population, and accentuated clients’ rights put pressure on reforming the health system. Second, the development of medical technology and information systems and restrictions due to economic constraints require reforms [3]. In addition, from the client-orientation viewpoint, frequent health and social care users would particularly benefit from integration of social and health services [3,4].

Integration has recognised effects on the delivery of high-quality care that is more cost-effective [6]. However, in the integration of health and social care, the starting point is the way cohesive or fragmented services are formed, specifically from a client’s individual care pathway point of view. Timeliness, individual need for services, client orientation, and seamlessness are the key drivers of integration [7]. However, numerous articles have addressed the challenges in creating integrated services [8]. Because integrated care is a multidimensional concept, the barriers and facilitators range from interaction of professionals to administration, organizational structures, and funding. Shared vision, joint planning of services, and aligned support from managers of collaborating work units are important elements in creating integration, and they have great importance in patient-centred care [8].

A need for a conceptual framework to understand integrated care and social services from a governance viewpoint is prevalent [6,9,10,11]. The World Health Organization has developed a conceptual framework for people-centred and integrated health care services. The framework presents individuals, families, and communities at its centre, which means that people-centred and integrated social and health service delivery must be supported by an enabling policy environment that promotes healthy public policies [12]. Managers of health and social care have a need for tools to assist integration by focusing and guiding integration efforts [13]. Organizational learning theory highlights that the ongoing learning capacity of governance strongly depends on top-level leaders’ behaviours, organizational structure, culture, and flexibility as well as uncertainties in the environment in which the organization functions [14,15]. Gaining consensus about the targets of integration and putting them into a strategic framework is important [6]. Because health and social care has fragmented into a complex system, the analysis of integration is difficult. [6,7].

Nicholsson and colleagues [6,10] identified a conceptual framework of integrated care consisting of ten health care governance elements of integration. We have applied those governance elements in this study (presented in the research methods chapter).

The general aim of the study is to assess the development of the governance of health and social care integration in Finland. First, we explore regional approaches to integrated care in Finland by using Nicholson et al.’s [6,10] conceptual framework. Second, we systematize regional approaches to integrated care, addressing both regular regions without integrated unified administration and regions with integrated unified administration as well as the ways the two different approaches support implementation of integration in the regions. Third, we evaluate how Nicholson et al.’s framework can be applied to an analysis of integrated governance.

### Health and social care system in Finland

In Finland, health and social services as well as most other welfare services, including schools, libraries, and children’s day care, are based on public funding and provision through the highly decentralised responsibility of the mostly small municipalities. The municipalities form joint municipal authorities to offer specialist services and, in case of smaller municipalities, for primary care and social services, too. To finance the services, the municipalities have the right to levy taxes. In addition, they obtain funding through state transfers and users’ fees. The Finnish health and social care system has been described in detail elsewhere [9,16,17,18,19,20].

In the statutory health care system, the country is divided into 20 hospital districts. The municipalities manage and fund the hospital districts within their respective catchment areas, and they are responsible for organizing and providing specialist medical services for the residents of member municipalities. Each district runs a regional hospital. Each hospital district belongs to one of the five university hospital catchment areas (UHCAs) led by the hospital districts associated with the medical schools in five major cities (Helsinki, Turku, Tampere, Oulu, and Kuopio). The UHCAs coordinate the provision of specialised medical care, information systems, medical rehabilitation, and procurement [16,18,19].

In terms of primary health care, municipal health centres provide a wide range of curative and preventive services, such as general practice consultations, maternity and child health clinics, dental health services, and mental health and substance abuse services. The health centres are managed by about 170 primary health care authorities, which are administratively either joint municipal bodies or single municipalities [9,16,19]. Most of the municipalities have merged the earlier separate administrations of health and social care services under one administration. The municipalities provide social and elderly care services that include social work and guidance, family counselling, social rehabilitation, and assisted living and home care for the elderly. In most of the municipalities, home nursing and home help services have been merged into home care.

Due to local arrangements, the health and social services in Finland are under two separate types of regional administration. The regular way to arrange services, which is defined in legislation, involves a separate authority for specialized health services and then several independent municipal authorities for primary health care and social services (which may or may not work in an integrated manner). The specialized health care authority is responsible for coordinating health services but how this is practiced varies between regions.

A new type of regional administration is a regional joint authority, which municipalities form through voluntary agreements. Currently, eight out 20 regions have a joint authority of this type. On average, these regions are smaller, and altogether they constitute around 20% of the Finnish population (Table 1). In these regions, all public specialized, primary and social care services are under one unified administration and management, which allows the integration of various aspects of services and other operations. Six out of eight of these joint authorities were founded after 2018.

**Table 1:** Unintegrated and fully integrated administrations of regional health and social care authorities in Finland.


REGULAR (MUNICIPAL) ADMINISTRATION (*N* = 12)	REGIONAL JOINT HEALTH AND SOCIAL CARE AUTHORITIES (*N* = 8)

Statutory Special care authority = hospital district = municipal federation Independent municipal authorities for primary and social care (practices for integration vary) Planning responsibility with hospital district (disease-based care pathways often applied)	Voluntary – agreement by the municipalities (est. 20% of the Finnish population) All public specialized, primary and social care under one administration (HR, financing). Potential for integrated planning and work practices Often-shared electronic client information system Six of these organisations were founded between 2018 and 2019


The municipalities and the health and social care authorities in Finland have developed their health and social services in extreme uncertainty for the last 15 years due to the government’s series of failed attempts to reform the health and social care system [4,5,7,16,20]. In general, government policies have adopted integration and regional administration, but the reform proposals have been quashed for legal and constitutional inconsistency related to complex and extensive legislative packages and political disputes on issues, such as the role of the private sector.

In terms of developing service arrangements and integrated care, the situation in each region has constantly changed, and the municipalities and the health and social authorities have adapted their operations to government policy processes and local circumstances, which has resulted in variations in the pace of reforms, such as implementing integrated care.

At the beginning in 2023, the so-called “well-being services counties” will take responsibility for organizing all health and social care services. After the reform, Finland will have 21 new well-being services counties. In addition, Helsinki will organize and continue to produce its own social and health care services in the future [21].

As in many other countries, the aging population in Finland significantly influences the development of health and social services. The life expectancy of the Finns at age 65 now exceeds 20 years. In 2017, 21% of the population was 65 and older. Three in five of those aged 65 or older reported having at least one chronic condition or disability, which is a higher proportion compared to other EU countries [19]. In addition, Finland is sparsely populated due to rural depopulation and internal migration to urban areas. The delayed system reform is an additional factor affecting the development of health and social services in Finland [16,19].

The general aim of the study is to assess governance of health and social care integration in Finland. First, we explore regional approaches to integrated care in Finland by using Nicholson et al.’s [6,10] conceptual framework. Second, we systematize regional approaches to integrated care, addressing both regular regions and regions with integrated unified administration as well as the ways the two approaches support implementation of integration in the regions. Third, we evaluate how Nicholson et al.’s framework can be applied to an analysis of social and health care governance.

## Research Methods

The study is a qualitative review that consists of two phases (Table 2) comprising administrative and planning documents on health and social services (*n* = 176) from the 20 regional care authorities and additional interviews of national health and social care evaluation officers (*n* = 5). The interviews were conducted via Microsoft Teams, and semi-structured discussion was based on the analysis carried out. The study analyses administrative and planning documents on health and social services from the 20 regions in Finland. The documents were collected in May–June 2019 and completed in 2020. The data included most recent documents from relevant periods, such as recent annual reports, organizational plans, agreements between authorities and municipalities, and regional plans for intended national health and social service reforms.

**Table 2:** Data and analysis process.


	PHASE 1	PHASE 2

**Aim**	Analyse of governance of health and social care integration in Finland. Evaluate how Nicholson et al.’s (2013) framework can be applied to an analysis of integrated health and social care governance in Finland.	

**Data**	Regional reports and documents pertaining to social and health care reform, other preparatory materials related to integrated care	Interviews of national health and social system evaluation officers (*n* = 5)

**Methods**	Qualitative deductive content analysis	Semi-structured interviews and mixed qualitative analysis

**Frame of analysis**	Elements for integrated health and social care governance (Nicholson et al. 2013)	

**Elements of integrated governance guiding the analysis**	**Joint planning:** Joint strategic needs assessment agreed; formalising relationships between stakeholders; joint boards; promotion of a community focus and organisational autonomy; guide for making decisions collectively; multi-level partnerships; focus on continuum of care with input from providers and users **Integrated information communication technology:** Systems designed to support shared clinical exchange (i.e., shared electronic health record; a tool for systems integration linking clinical processes, outcomes, and financial measures) **Change management:** Managed locally; committed resources; strategies to manage change and align organisational cultural values; executive and clinical leadership; vision; commitment at meso- and micro-levels **Shared health and social care priorities:** Agreed target areas for redesign; role of multidisciplinary health and social care networks and/or panels; pathways across the continuum **Incentives:** Incentives provided to strengthen care co-ordination, such as pooling multiple funding streams and incentive structures (e.g., equitable funding distribution); incentives for innovation and development of alternative models **Population focus:** Geographical population health focus **Measurement – using data as a quality improvement tool:** Shared clinical population data to use for planning and measurement of utilisation focusing on quality improvement and redesign; collaborative approach to measuring performance provides transparency across organisational boundaries **Ongoing professional development supporting the value of joint working:** Inter-professional and inter-organisational learning opportunities provide training to support new methods and align cultures; clearly identifying roles and responsibilities and guidelines across the continuum **Client/community engagement:** Involve patient and community participation by use of patient narratives of experience and wider community engagement **Innovation:** Resources are available and innovative models of care are supported.	

**Level of maturity**	Demonstrated (4) = in strategy and practice in the whole region Demonstrated (limited) (3) = in strategy and practice partly or in some municipalities Developed (2) = planned for implementation in the whole region Developed, limited (1) = planned for partial implementation or in some municipalities Nil (0) = none	

**Outcome**	Descriptive analysis of integrated social and health care governance in Finland Evaluation of the feasibility of the integrated elements (Nicholson et al. 2013) for analysis of integrated social and health care governance	


In the first phase, we analysed the documents using deductive content analysis in terms of the governance elements supporting integrated care. We adapted the frame of analysis from Nicholson et al. [6,10], who identified 10 key elements of integrated health care governance through an extensive review of earlier research on governance models in integrated care settings. We modified the application of the elements (Table 2) by adding social services in the analysis of integration because this is a major issue in the development of the Finnish health and social service systems.

According to the levels, the presentation of the key elements in each document was classified into a level ranging from 0 to 4, using scale of half decimals. This was based on a choice made by the four researchers. We identified the descriptions of the elements in the regional planning and administrative documents and categorised the regions for each element into four levels according to the scale given in Table 2. The lowest level (0) for the element indicated that we were not able to distinguish any reference to the element in the documents of the region. The highest level (4) corresponded to the finding that the element was clearly mentioned in the region’s strategy documents, and it was implemented in governance throughout the regional services. In some cases, when the regional implementation of the elements was not possible to rate according to the integer scale, we also allowed the use of half-grade ratings, based on the collective decision of three of the four researchers.

In the second phase, we compared our findings on the identification of the governance elements by interviewing the five evaluation officers responsible for regional assessments within the national annual health care and social services evaluation. The purpose of the supplementary interviews was to compare the consistency of the results with the views of evaluation officers who have deep knowledge of the regions. The annual evaluation is carried out by the Finnish Institute for Health and Welfare and is coordinated regionally by the evaluation officers. Rissanen et al. [22] described the evaluation process and methodology in detail.

## Results

### Elements of integrated care governance

The most advanced elements of integrated care governance in the Finnish health and social care system were *joint planning, shared health and social care priorities*, and *client/community engagement*. In addition to these elements, *measurement of data* and *continuing professional development* reached at least Level 2 (developed) in all regions. Of the ten elements, *information communication technology, change management, population focus*, and *innovations* showed differences between regions with the regular and unified integrated administration.

Regions with unified regional administration have joint boards and work groups of stakeholders, who implement strategy in action. Regions with regular administration have so far planned a common strategy, but the municipal authorities have the right to make independent decisions.

The modelling of the service paths between primary and specialized care has been piloted in most regions. For instance, in North Savo Health Care District, some services have functioned seamlessly for a long time between primary and special health care and between different sectors (e.g., substance abuse services and mental health services). In Satakunta Health Care District, modelling of the service paths and descriptions of the service processes has been planned in Jyväskylä, the central city of Central Finland district, population and officials had to wait until the brand-new central hospital (NOVA) was made ready. New facilities have been constructed without borders between specialized and primary health care. In South Karelia Health and Social Care Region, all services for children and young people have been organized in a new way. Services for children and young people from specialized care, primary care, and social services are organized under joint management. Similarly, in North Karelia, health and social services are organized jointly in health and social centres’ multi-professional teams (which have been developed nationally at this stage). In these integrated joint authorities, one management team plans the integrated services instead of 15–20 municipalities and a hospital.

A wide range of instruments has been used for quality improvement. Typically, information concerning client satisfaction (e.g., patient-reported experience measures) is gathered in every region, but in many cases with different methods. The degree to which the quality information has been used across the borders of sectors to foster integration is difficult to determine due to missing information in documents.

In Finland, requirements for the formal education of the health and social care staff are high. Much training occurred, but the documents do not reveal the degree to which interprofessional and intersectoral training has been arranged. There are mentions, such as “common training” in South Ostrobotnia and North Karelia and “partly multiprofessional” training in Kanta-Häme.

Although most elements of overall integrated care governance were advanced to at least some extent, *incentives* were not recognised as having been used in supporting integrated care in any region. As stated above, health and social services in Finland are mainly funded and provided by the public sector and the professionals are mainly salaried municipal sector employees. Health and social care authorities may use incentives for employees but often it is done for purposes other than care integration, such as supporting recruitment.

The national evaluation officers mostly confirmed our findings based on their expertise, and the differences between grades formed by researchers and the evaluation officers’ views were minor. Main discrepancies involved the extent of implementation of individual governance elements that had been insufficiently described or recognised in the regional documents.

Due to the planned national health and social care system reform, the structures of *joint planning* of the integration of services have been under construction in all regions. Regarding *integrated information communication technology*, the advantages of integrative information systems are also widely recognized. In most regions, however, the health and social care client-record systems are fragmented (e.g., each municipality may have a system, while health and social services operate separate systems as well). Almost all regions having the regional joint authority also had a unified information system in health care (the same system in primary and secondary care), and some had a unified system in health and social care, even though at the time of the study the client records could not be used across health and social care due to legal restrictions.

On average, the regions with integrated regional administration had higher grades in terms of implementing the different elements of integrated care governance, but for most elements, the difference was minor. In addition to the governance elements of *joint planning* and *integrated information communication technology*, the integrated care authorities made a difference to the regions with regular administration for the element of *change management* (e.g., shared vision). Only two of the integrated care authorities had been launched before 2019, which probably explains why integrated care governance elements had not yet strengthened in these newly established authorities. However, the region of South Karelia, which was established in 2010, received the highest grades for all the elements of integrated care governance (i.e., in strategy and practice in the whole region or at least in some municipalities; see also Figure 2), excluding *client/community engagement*. Strengthening of the client/customer engagement (e.g., panels of clients and so-called “experts of their own experiences”) has been widely acknowledged and already demonstrated in most regions.

**Figure 1:**
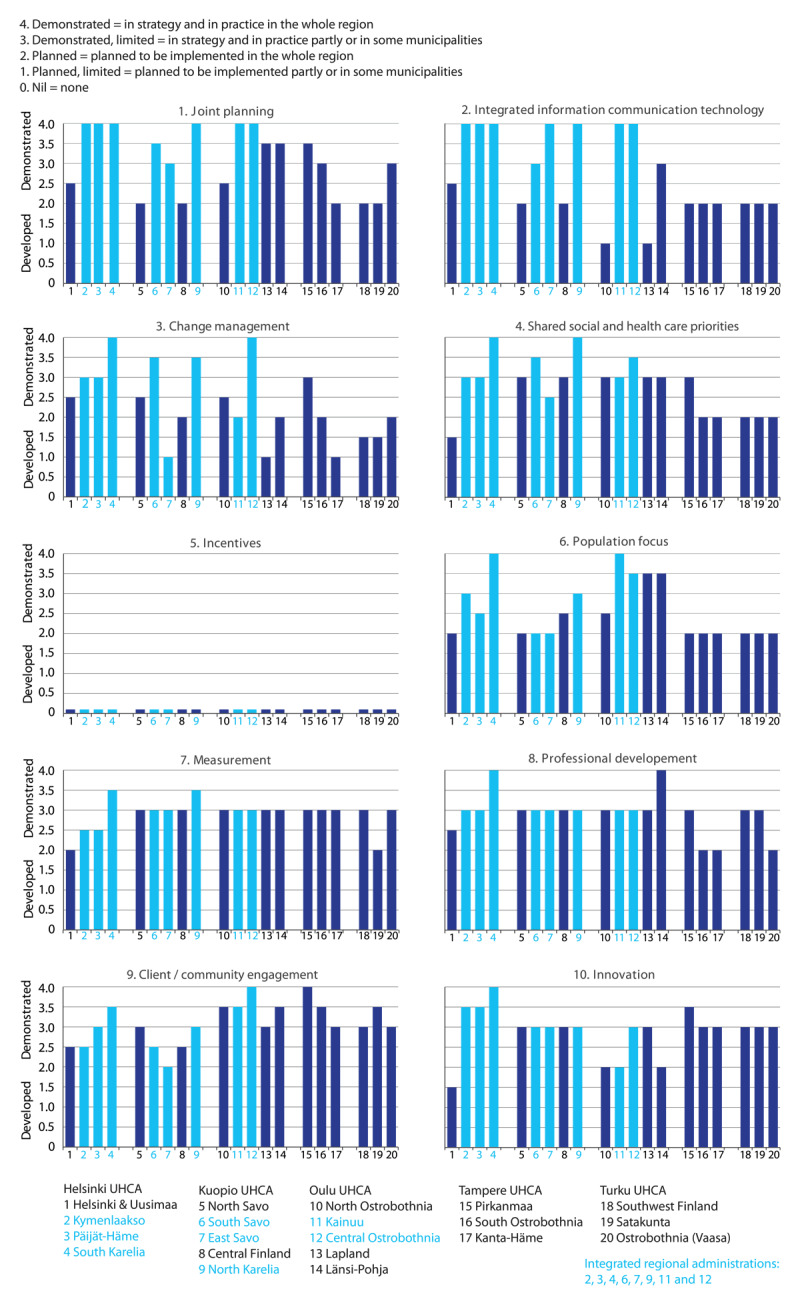
The level of integrated governance elements in different regions in Finland. (The light-blue columns indicate joint authority administrations; dark-blue columns indicate regular [municipal] authorities).

In Figure 1, the regional advance of integration is described in terms of the ten elements of integrated care governance. The light blue columns are intended to demonstrate joint authority administrations and correspondingly dark blue columns demonstrate regular (municipal) authorities.

**Figure 2:**
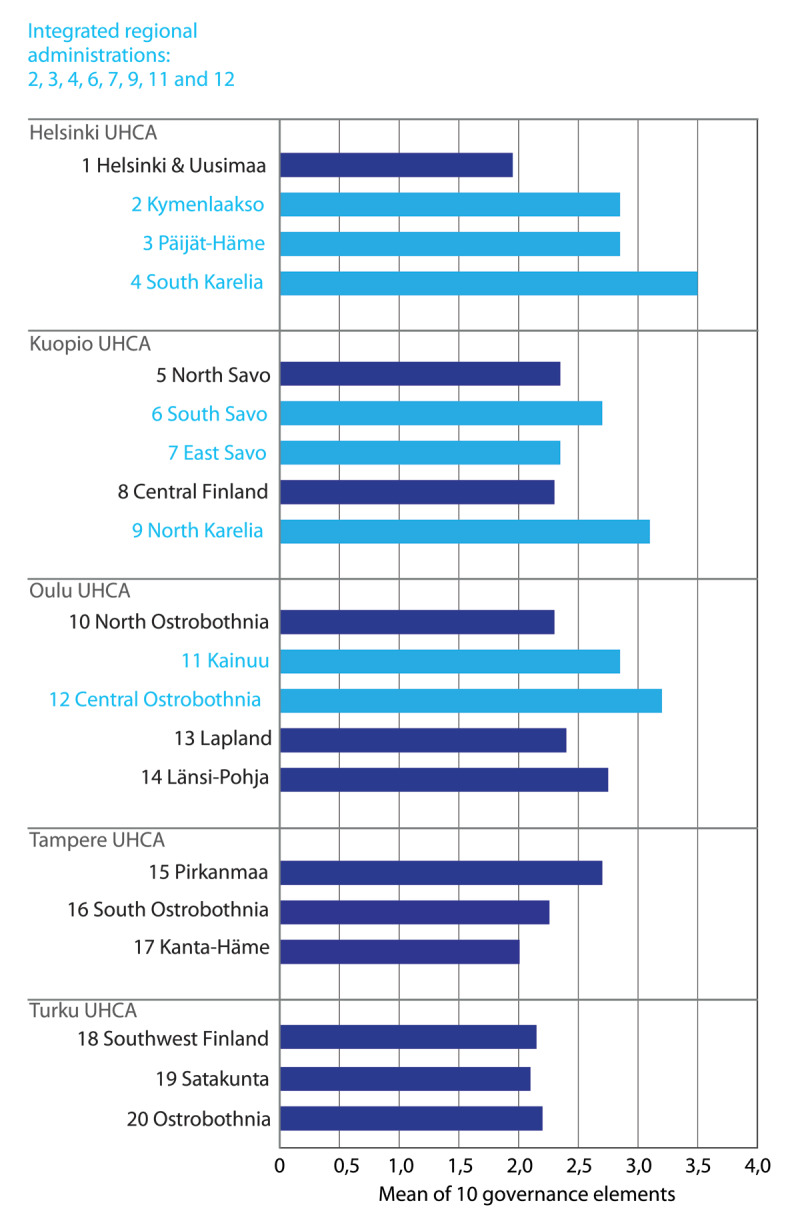
Overall picture of integrated social and health care governance in Finland. (The light-blue columns indicate joint authority administrations; dark-blue columns indicate regular [municipal] authorities).

Figure 2 shows the mean for the ten integrated governance elements in both joint authority administration and regular (municipal) authorities in Finland. In general, the regions with joint regional administration have higher grades for the integrated governance elements. The highest summary measure was assigned to the South Karelia region, which was established in 2010 as the first joint social and health care authority. However, some regions with only recently established joint regional administration have also proceeded well with integrated care governance of health and social services.

## Discussion

This study gives a picture of integrated health and social care governance in Finland in different administrations. In general, integrated care is already relatively well advanced in Finnish health and social care. Because local governments, municipalities, and municipal joint organisations run public health and social services, collaboration across sector borders has been relatively uncomplicated to organise. Still, challenges to organising co-operation and coordination between health and social care as well as between primary care and secondary and specialized care have arisen, because municipalities organise the first and hospital districts the latter. In recent years, in eight of 20 regions, the municipalities have voluntarily established the joint health and social care authorities running all health and social services under a single administration [16,17,18,19,20]. Now the regions are under pressure to promote integration because the well-being service counties will begin from the beginning of 2023.

Our results strongly suggest that the integrated organizations, joint management, and funding in the new joint authorities have an effect on integration. In the integrated joint authorities’ joint planning, building shared priorities, change management, and integrated information systems were at a higher level. It is possible that building a joint strategy, shared values, unified organizational culture, and practices are easier when the management is under one executive team instead of executive teams of several municipalities and one hospital district, which is the case in the regions with regular administration. However, in several regions, despite the separate management groups, the integrated governance elements seemed to be well developed. However, because the analysis in the study is based on documents analysed using Nicholsson et al.’s [6,10] framework and supplemented by evaluation officers’ viewpoints, it needs to be noted that we do not have a picture of the way clients experience the integration in their service paths.

According to our experience, extending the presented ten elements to an analysis of the integration of social services with specialized and primary health care was feasible. In terms of a more granular analysis of elements of governance that support integrated care [16,17], half of the ten elements were successful or well advanced in all regions. These elements were *joint planning, population focus, measurement of data, continuing professional development*, and *client/community engagement*. In our study, these five elements at least reach the level of “planned to be implemented in the whole region” in all regions. Several factors have contributed to this. Public health and social services funded and provided by municipalities and joint municipal organizations have obviously supported integration. In addition, government policies and reform plans have subscribed to integrated care in recent years [4,18,22]. However, substantial variations in approaches to and pace of development of integrated care exist across regions. The variation of the integrated governance elements we analysed was particularly high with *integrated information communication technology* and *change management*. In part, this may be because in a document-based analysis, the elements of change management in health and social care planning documents are challenging to identify and the ways to describe these elements in documents vary regionally. The study confirms the contributions of previous studies that showed multiple elements are required to ensure successful and sustainable integration efforts. [6,23,24].

The interviews of the national evaluation officers confirmed our findings. The agreement was quite high. The main differences emerged over whether the district had planned to implement the element in question or whether the element was already implemented in some parts of the region. Care integration was often at a very high level in some municipalities, especially in the regions with traditional administration, but development was weaker in other municipalities. Although it is possible to analyse integration based on a specific theoretical model, deep knowledge of the region is highly important to draw an analysis of the level of integration. Nicholsson et al.’s theory has been applied in the Australian [10] health system in addition to the current study on the Finnish social and health system.

In summary, integrated care has been planned but implementation is partly fragmented and is dependent on regional features such as financial resources, aging of the population, and regional politics. However, integrated administration of health and social services does not determine how the integration has been developed in the regions, but it must be noted that the regions with integrated administration are represented by rather young organizations. In general, the integration has placed more focused on integrating primary care and specialised health care, rather than integration between health and social services. In the regions with integrated administration, integration between health and social services has seen greater development.

In Finland, as in many other countries, a clear driver of integrated care is cost containment [6]. In this context, the aging population seems to be a major factor; for instance, Eastern Finland, where many regions with integrated health and social care authorities are located, also has the highest regional share of people older than 75. In addition to cost savings, in newly established joint authorities, an important goal seems to be to create transparent and clear structures that strengthen governance that supports integration.

Promoting integration should be inclusive, and thus clients should be more strongly integrated into the development of operating models of integrated care to promote quality, efficiency, client-oriented culture and cost-effectiveness.

The key findings of this study confirm that more research on social and health care governance integration and its measurement is needed so that countries and regions with various kind of social and health care systems can be measured, analysed, and studied to understand the current state of integration. Suitable models and indicators need to be developed so that integration can be benchmarked at the national and international levels. Integration is a matter of coordinated and seamless service paths, which is likely of interest regardless of the system, region, or country. Integration or lack thereof can have a very large influence on the success of services that clients need.

Clients who require multidisciplinary services often use both social and health care services, and thus integration is more important from orientation, effectiveness, and cost viewpoints. Integrated governance creates the basis for the implementation of practical integration between social and health care. However, integrated governance also lays the foundation for an operating culture, when social and health care organization has a common shared interest and values in promoting the integration. Integration is at its best an automation tool for service coordination.

Our study has some limitations. First, because regional planning in Finnish health and social care primarily focuses on health services, our analyses based on regional administrative and planning documents have probably been less detailed on integration comprising social services. Second, the quality of documents varied across regions. Some documents seemed to be quite positive concerning integrative care in the region. The descriptions concerning the state of affairs may not be as objective as we could expect. Moreover, some elements of governance of integration were not described at all in the documents or in a very narrow way. Third, in our study we were not able to investigate the actual implementation of integrated care.

## Conclusions

The study showed that integration of social and health care consists of several separate elements that are closely connected to each other [6,10]. Their definition is necessary to make it possible to measure and evaluate integration. Recognizing the connection between elements reinforces an understanding of the deeper dimension of integrated governance. The result of this study draws attention to integrated governance of social and health services, which should be strengthened more broadly in multidisciplinary cooperation [3,24]. As a conclusion, the constant change and the pursuit of administrative coherence should be considered when analysing implementation of integrated care. It is possible that the picture of integration we have developed is not highly precise. However, at face value, it appears credible.

Overall, our approach as such has proven feasible and the deductive content analysis carried out allowed us to identify various elements for integrated care governance. Consequently, it seems feasible to use elements of integrated care governance to monitor development of integrated care in regions in a way that is more structured. The analysis also provides an overview at the national level.

In further research, based on this analytical integrated governance framework, it is possible to apply an assessment and monitoring tool for to measure the reform of government health and social care as well as regional development projects relative to the progress of integrated care.
